# Quantification of β-Elemene by GC–MS and Preliminary Evaluation of Its Relationship With Antitumor Efficacy in Cancer Patients

**DOI:** 10.1155/jamc/6694947

**Published:** 2025-03-30

**Authors:** Juanjuan Hou, Jia Yi, Yan Wang, Lili Cui, Wenwen Xia, Zhengyan Liang, Liya Ye, Zhipeng Wang, Shouhong Gao, Zhan Wang

**Affiliations:** ^1^College of Traditional Chinese Medicine, Yunnan University of Traditional Chinese Medicine, Kunming 650500, Yunnan, China; ^2^Department of Pharmacy, Second Affiliated Hospital of Naval Medical University, Shanghai 200003, China; ^3^Department of Oncology, Second Affiliated Hospital of Naval Medical University, Shanghai 200003, China

**Keywords:** β-elemene, exposure level, GC–MS, plasma, therapeutic drug monitoring

## Abstract

**Objectives:** To establish and validate a sensitive and robust gas chromatography–mass spectrometry (GC–MS) method for the quantification of β-elemene in human plasma and assess the correlation between antitumor effect and β-elemene concentration *in vivo*.

**Methods:** The chromatographic column was HP-5 ms (30 m × 0.25 mm, 0.25 μm, Agilent, United States of America). The carrier gas was helium (purity > 99.5%). The flow rate was 1.0 mL/min and the total run time was 11.0 min. The plasma sample was pretreated with protein precipitation plus liquid–liquid extraction. Cancer patients were enrolled and their samples were collected for analysis.

**Results:** Calibration range of β-elemene was 200.0–20,000.0 ng/mL, with correlation coefficients > 0.99. The intra- and interday precision and accuracy were less than 5.8% and within the range of −10.4%–6.6%. The exposure level of β-elemene in the responder group ranged from 278.13 to 11,886.27 ng/mL, with a median of 3568.91 ng/mL, while in the nonresponder group, the range was from 675.92 to 9716.52 ng/mL, with a median of 3351.94 ng/mL. No difference was found in the β-elemene exposure level between the two groups (*p* > 0.05).

**Conclusions:** This method was effectively developed, validated, and utilized to quantify β-elemene in cancer patients. The initial findings indicated no significant relationship between therapeutic efficacy and the concentration of β-elemene.

## 1. Introduction

Cancer ranks among the foremost contributors to premature mortality, following cardiovascular diseases, across 57 nations [1]. In 2050, there will be a remarkable 76.6% rise in newly diagnosed cancer cases and an 89.7% increase in cancer-related mortality worldwide compared to 2022. The age-standardized incidence and mortality rate for breast, prostate, lung, and colorectal cancers in the top 10 countries surpassed the global averages [2]. The primary therapeutic methods for cancer include radiotherapy, chemotherapy, targeted therapy, and immunotherapy. For patients with intermediate to advanced stages of cancer, these treatment methods have shown short-term efficacy but are accompanied by a series of adverse reactions (ADRs) that significantly hinder their clinical application [3]. Many studies have substantiated the roles of herbal remedies in augmenting pharmacological effects for cancer, aiming to mitigate ADRs and enhance therapeutic efficacy [4–8].

β-elemene is a bioactive compound isolated from the Chinese herb *Curcuma longa*, which has a wide range of anticancer effects and can be used in many cancer types including colorectal cancer [9, 10]. The β-elemene emulsion injection was approved as a new Type II anticancer drug by the drug regulatory authority under the Chinese Ministry of Health (currently the National Medical Products Administration) in 1995 [11]. Currently, β-elemene injection, β-elemene milk injection, lyophilized powder injection, and aerosol have been developed and applied in clinical practice, usually in combination with chemotherapy drugs. The β-elemene injection has noncytotoxic and broad-spectrum antitumor characteristics compared to traditional chemical antitumor drugs, and it is used in China for the treatment of cancers, for instance, lung cancer, leukemia, breast cancer, liver cancer, etc [12–15]. β-elemene has demonstrated significant antitumor efficacy in both *in vivo* and *in vitro* studies, and its underlying antitumor mechanisms are progressively being elucidated [16–20]. A study involving 102 patients with squamous cell carcinoma in the esophagus compared pathological parameters and outcomes of patients treated with and without β-elemene therapy and demonstrated that β-elemene prolonged 3-year overall survival and progression-free survival [21]. Furthermore, β-elemene could enhance the efficacy of lung cancer treatment and extend patient survival [22, 23]. When combined with immunotherapy, β-elemene exhibited a synergistic effect in the treatment of colorectal cancer [24, 25]. Nevertheless, not all cancer patients will benefit from the β-elemene. The therapeutic effect of the drug is closely related to the *in vivo* exposure level of its metabolites, but there is a lack of research evaluating the correlation between efficacy/ADR and the exposure level of β-elemene.

Many quality control (QC) methods for β-elemene formulations, such as gas chromatography–mass spectrometry (GC–MS) methods, have been established; however, these methods have primarily been applied for *in vitro* analysis of β-elemene. Li et al. reported a GC–MS method for seven volatile constituents including β-elemene in rats, and a pharmacokinetic study was performed [26]; Zhu et al. quantified the β-elemene in plasma after oral administration of cablin essential oil extract to rats by the GC–MS method [27]. However, the above two studies did not detect the concentration of β-elemene in human plasma. Lv et al. developed an ultra-performance liquid chromatography–quadrupole time-of-flight/mass spectrometry method for the determination of turmeric rhizome extracts, including β-elemene [28], but it did not allow for the accurate quantification of β-elemene. Li et al. developed an GC–MS method for the determination of β-elemene in plasma [29], but the method necessitates a substantial volume of plasma samples and an intricate sample processing protocol. Nevertheless, the limited sensitivity of these methods, the substantial sample size necessary for analysis, and the lack of comprehensive validation and testing in human blood specimens have impeded their clinical application. Most importantly, the relationship between the exposure level of β-elemene and its treatment outcomes has not been analyzed.

The aim of this study was to develop a simple, rapid and sensitive GC–MS method for the determination of β-elemene level in human plasma, and then the association of exposure level of β-elemene and its treatment efficacy was assessed in 73 cancer patients treated with β-elemene for the first time.

## 2. Materials and Methods

### 2.1. Chemicals and Reagents

**Figure 1:**
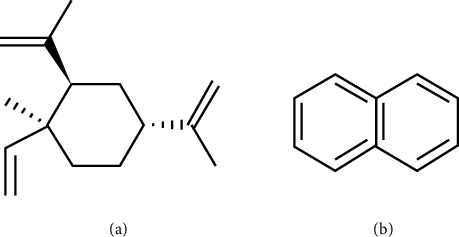
Chemical structures of β-elemene and IS. (a) β-elemene. (b) Naphthalene.

The standards of β-elemene (Lot: J14GB154783) (purity: > 98.0%) and naphthalene (Lot: Y01S7C20290) (internal standard [IS]) (purity: > 98.0%) (Figure 1) were supplied by Shanghai Yuanye Bio-Technology Co., Ltd. Mass spectrometry–grade acetonitrile and methanol were purchased from Merck (Darmstadt, Germany); ultrapurified water was provided by Watson (Shenzhen, China). Human blank plasma containing EDTA-3K anticoagulant was donated by Shanghai Changzheng Hospital (Shanghai, China).

### 2.2. GC–MS Instrumentation and Conditions

The GC–MS system was comprised of an Agilent 7890A-5975C, integrated with an Agilent 7693 autosampler and an electron impact ionization (EI) source. For chromatographic separation of the analyte, an Agilent HP-5 ms capillary column (30 m × 0.25 mm, 0.25 μm) was used to complete the separation. The temperature program was initiated at 60°C, maintained for 3 min, followed by a ramp to 160°C at a rate of 50°C/min and held for an additional 3 min; the temperature was subsequently increased to 280°C at the same rate, with a final hold of 5 min. High-purity helium (> 99.5%), which served as the carrier gas, was delivered at a constant flow rate of 1 mL/min. The injector and transfer line temperatures were set to 280°C and 290°C, respectively, while the inlet temperature was maintained at 280°C. A 1 μL aliquot of sample was taken with a split ratio of 1:2 for analysis. For mass spectrometric detection, the EI source operated at an ionization energy of 70 eV, with a mass spectral scanning range of 50–600 m/z. The detection ions selected for β-elemene and the IS were m/z 93 and m/z 128, respectively.

### 2.3. Preparation of Stock and Working Solutions

Stock solutions of β-elemene and IS were prepared separately in methanol both at final concentrations of 1.11 mg/mL. The stock solutions were aliquoted and stored at −80°C. The working solutions of β-elemene were prepared freshly by diluting the stock solution with 10% methanol aqueous solution. Calibration standards were prepared by taking 100 μL of each working solution and then adding 900 μL of blank plasma (1:9, V:V). The concentrations of calibration standards for β-elemene were as follows: 200.0, 400.0, 1000.0, 2000.0, 4000.0, 8000.0, 16,000.0, and 20,000.0 ng/mL. QC samples were weighed and prepared separately in the same manner and diluted with blank plasma to obtain 400.0, 4000.0, and 16,000.0 ng/mL QC samples, which were placed in Eppendorf tubes and stored at −80°C until use.

### 2.4. Sample Pretreatment

Protein precipitation combined with liquid–liquid extraction was used to extract β-elemene from plasma. For 100 μL aliquot of plasma sample, 10 μL of IS solution (10 μg/mL, freshly prepared) was added, vortex-mixed for 1 min, and then the mixture was quenched with 200 μL of precooled(4°C) acetonitrile and 20 μL of saline, and vortex-mixed again for 30 s prior to centrifuging for 10 min at 4°C at 13,000 × g. After centrifugation, all the supernatant was collected and extracted with 100 μL of hexane. After vortex-mixing and centrifugation again using the same conditions, a 10 μL aliquot of hexane was then injected into the GC–MS system for analysis.

### 2.5. Human Sample Collection

Patients diagnosed with cancer who received adjuvant therapy involving inβ-elemene emulsion injection were enrolled from December 10, 2022, to December 10, 2023, at the Second Hospital of Naval Medical University (Shanghai Changzheng Hospital). The experimental protocol underwent review and received approval from the hospital, with all participants providing informed consent. Inclusion criteria encompassed the followings: (1) confirmed cancer diagnosis, (2) age ranges from 18 to 75 years old, (3) Eastern Cooperative Oncology Group (ECOG) performance status (PS) scores between 0 and 2, (4) expected lifespan exceeding 6 months, (5) absence of significant liver and renal function abnormalities in routine assessment, (6) patients undergoing therapy including β-elemene, and (7) a minimum of 2 consecutive courses of β-elemene prescribed. Exclusion criteria included the followings: (1) pregnant or breastfeeding patients; (2) patients with cardiac, psychiatric, or other severe comorbidities; (3) allergic to β-elemene; (4) patients with extensive visceral metastases; (5) patients with hematologic malignancies; and (6) β-elemene administration of less than 2 consecutive courses. Blood samples were collected immediately following the intravenous administration of β-elemene into EDTA-3K tubes, and the samples were centrifuged for 10 min at 4500 × g at room temperature, after which the supernatant was transferred into cryogenic tubes. The samples were preserved in a refrigerator at −80°C until analysis. Patients were followed up for treatment efficacy and ADR. Treatment efficacy was categorized as complete response (CR), partial response (PR), stable disease (SD), or progressive disease (PD).

### 2.6. Method Validation

Method validation was completed under the guidance of Chinese Pharmacopoeia (2020 edition) and FDA guidelines. The developed method was validated in terms of specificity, linearity, inter- and intraday accuracy and precision, extraction recovery and matrix effect, stability, and carryover [30–32]. A detailed description of the method validation can be retrieved from the Supporting materials (available here).

## 3. Results and Discussion

### 3.1. GC–MS Condition Optimization

Based on an Agilent 7890A-5975C GC–MS system, the β-elemene was completely separated from the endogenous interferents with a symmetrical peak shape. Through a series of experiments, we chose the HB-5 ms high-resolution capillary column, which was suitable for the separation of trace metabolites in highly complex biological samples due to its excellent separation efficacy and peak symmetry. In setting the carrier gas flow rate, constant temperature nitrogen was used as the carrier gas and debugged between 1.0 and 1.5 mL/min, and finally, 1.0 mL/min was determined to be the optimal flow rate to ensure the best balance between analysis time and peak resolution. Temperature was another key parameter affecting the efficiency of GC–MS analysis. After several times of attempts, the temperature of the injection port was finally set to 280°C to avoid decomposition or adsorption of the active ingredients during the injection process and to ensure the complete volatilization of the sample. The sample volume was 1 μL, and the split ratio was 1:2. The detector temperature was adjusted to 280°C to improve the sensitivity and quality of signal acquisition. To prevent sample decomposition and congestion, the temperature of the transfer line was set at 290°C. Optimization of the programmed temperature's increased rate showed that maintaining the initial temperature at 60°C for 3 min and then increasing the temperature to 160°C at a rate of 50°C/min could achieve the desired separation in the shortest analysis time. In addition, in order to further improve the sensitivity of the analysis, the ionization energy mode of the GC–MS was adjusted, and the optimization results revealed that the limits of detection (LODs) and limits of quantification (LOQs) of β-elemene were significantly improved by tuning the collision energy to 70 eV and employing the SIM mode. The system was calibrated before and after each analysis with standards and QC samples.

### 3.2. Sample Pretreatment

Due to the complexity of the plasma matrix, sample pretreatment was often required to remove proteins and potential interferences prior to GC–MS analysis. To reduce proteins and other macromolecules in plasma, we tried to add different ratios of methanol and acetonitrile for precipitation (V: V, 1:2, 1:3, 1:4, and 1:5), but the protein precipitation brought very low and yet steady recovery of β-elemene (recovery < 10% and RSD% < 5%). The low solubility of β-elemene in methanol and acetonitrile may cause the extremely low recovery of the protein precipitation method, as the Log *p* value of it was 4.74. Then, we tested the Waters Ostro TM plates and Waters Oasis HLB SPE plates, which gave extraction recoveries of up to 40% but at a higher cost and unsteady recovery (RSD% > 30%), which may be explained by the plastic wall absorbance by the SPE plates. Considering the low but steady recovery obtained after the protein precipitation, we conducted liquid–liquid extraction using ethyl acetate, etc. of the supernatant; however, it was inadequate in fully eliminating the interference from endogenous plasma constituents that impact the analytes. Furthermore, ethyl acetate poses a potential hazard of causing irritation to the mucous membranes of the operator. Finally, we optimized the liquid–liquid extraction solvent and the procedures, and 200 μL of ice-cold acetonitrile and 20 μL of saline were added to 100 μL of the plasma sample, and the proteins were precipitated and effectively removed by centrifugation at 13,000 × g for 10 min at 4°C; all the supernatant was then extracted with 100 μL of hexane to further reduce matrix effects and improve the recovery of β-elemene. The optimization of this pretreatment method achieved high and steady recoveries (recovery > 87% and RSD% < 3.94%), providing high-quality samples for subsequent quantitative analysis, in contrast to other sample processing techniques [26, 27, 29]; in addition, we minimized the volume of plasma samples to 100 μL and implemented a more straightforward sample extraction approach that eliminated the need for evaporation and redissolution, thereby streamlining the operational procedures.

### 3.3. Method Validation

#### 3.3.1. Specificity and Carryover

**Figure 2:**
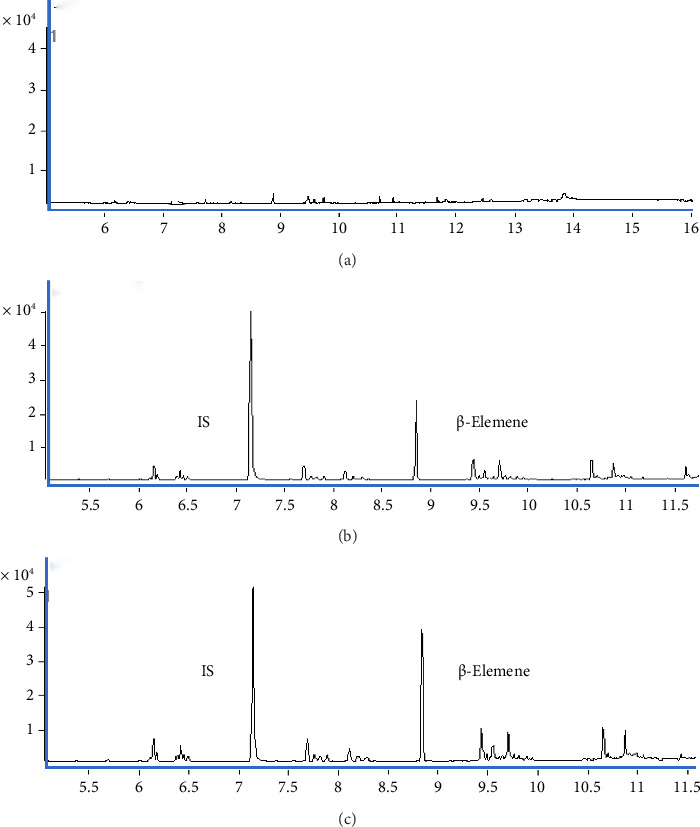
Comparison of chromatograms revealed no interferences to β-elemene and IS. (a) Blank sample. (b) Spiked plasma sample. (c) Real sample from one patient.

The retention times of β-elemene and the IS were about 8.8 min and 7.2 min, respectively, and both of them were well separated with no obvious interfering components in their retention times. Six batches of blank samples (Figure 2(a)), spiked samples (Figure 2(b)), and real samples (Figure 2(c)) were evaluated for specificity. The results showed that there were no interferences in the retention times of β-elemene and IS, and the retention times of the measured real samples were the same as those of the spiked samples, so the method met the specificity requirements.

**Figure 3:**
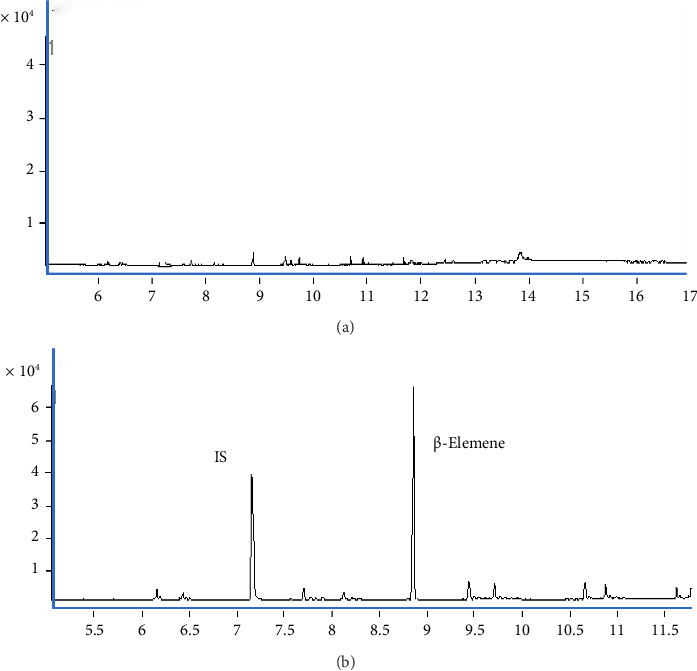
Typical chromatographic results of carryover of β-elemene. (a) Blank matrix. (b) Highest calibration standard.

The carryover of the method was estimated by injecting the highest calibration standard prior to the blank matrix for three cycles, and no β-elemene residue was found in the blank matrix, which met the requirement of biological sample analysis (Figure 3).

#### 3.3.2. Linearity and LLOQ

For β-elemene, 8 calibration standards were prepared and the calibration curve was obtained with weighting factors 1/*χ*^2^. The correlation coefficient *R*^2^ of the linear model of β-elemene was more than 0.99, and the typical linear regression equation of β-elemene was *y*=2.7653∗*x*+130.89, with the linear range of 200.0–20,000.0 ng/mL (Table 1). The LLOQ for β-elemene was 200.00 ng/mL. The backcalculated deviations of all calibration standards were within ±15% (±20% for LLOQ), in accordance with the requirements of pharmacopoeia.

#### 3.3.3. Inter- and Intraday Precision and Accuracy

In this experiment, by examining the inter- and intraday accuracy and precision of QC samples at low, medium, and high concentration levels and LLOQ, the results showed that the intraday accuracy of β-elemene was in the range of −10.38% to −3.75% with RSD% between 0.6% and 1.9%, and the interday accuracy ranged from −6.60% to −3.35% with RSD% between 1.0% and 5.8%, which all met the requirements (Table 2).

#### 3.3.4. Matrix Effect and Recovery

The endogenous substances in plasma were removed by a combination of protein precipitation and liquid–liquid extraction, and β-elemene and the IS were extracted to a great extent. The extraction recovery of β-elemene was in the range of 87.95%–96.25%, and the matrix effect was from 98.41% to 107.48%, with RSD% of recovery of less than 3.94% and RSD% of matrix effect less than 1.88% (Table 3). The results indicated that the combination of protein precipitation and the liquid–liquid extraction method resulted in high extraction recovery and the steady matrix effect compared to other studies [26, 27, 29].

#### 3.3.5. Stability

The stability of β-elemene was assessed at three concentration levels (low, medium, and high) including room temperature stability, short-term stability, long-term stability, and three freeze–thaw cycles. A significant decrease of β-elemene (short-term stability) was observed at 24 h, and this decrease was repeated in the second freeze–thaw cycle and in the long-term stability assessment (3 months). Finally, the results proved that the analyte was stable on the bench for 6 h, in the autosampler (4°C) for 6 h, and in the refrigerator (−80°C) for 1 month. These results claimed a rapid pretreatment procedure and measurements of β-elemene in the plasma matrix (Table 4).

### 3.4. Assessment of the Exposure–Effect Relationship of β-Elemene

**Figure 4:**
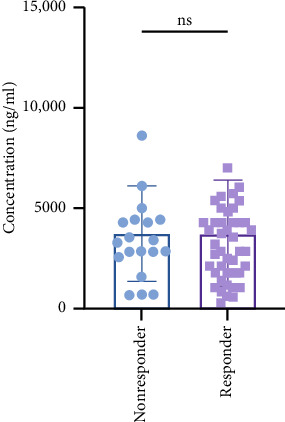
Comparative results showed that there was no difference in the concentration of β-elemene between the nonresponder and responder groups (*p*=0.97).

Eventually 73 patients including 51 males and 22 females were included, and their ages were between 26 and 79 years; we collected the basic information about the patient prior to the medication (Table 5). The treatment regimen mainly (30 patients) consisted of chemotherapy + targeted drug, followed by 17 patients whose regimen included radiotherapy + β-elemene. The highest number of patients were diagnosed with colon (24 patients) and rectal (18 patients) cancers. Upon completion of the follow-up period, it was noted that one patient had died of cancer, while the remaining patients had finished their treatment courses with varying outcomes: 19 experienced PD, 49 had SD, and 4 achieved partial remission (PR). In the analysis, we designated the PD as the nonresponder group, and SD and PR as the β-elemene responder group. This method was applied to determine plasma samples from 73 patients after administration of the drug. β-elemene plasma concentrations in the responder group ranged from 278.13 to 11,886.27 ng/mL, with the median of 3568.91 ng/mL, while in the nonresponder group, it was 675.92–9716.52 ng/mL, and the median was 3351.94 ng/mL (Figure 4). These results suggested a huge interindividual variation in the level of drug exposure, possibly due to the rapid metabolism of β-elemene. Based on the *in vivo* pharmacokinetic study of β-elemene in rats, the half-life (T1/2z) of β-elemene was found to be only 15.964 ± 1.938 min and the clearance (CLz) was found to be 0.014 ± 0.003 L/min/kg [33]. The elimination half-life (T1/2z) of β-elemene in humans ranges from 1.91 to 2.41 h, with a systemic CLz of 0.54–0.68 L/kg/h [29]. Chen et al. found that the human plasma concentrations (*C*_max_) were 7.6 ± 4.7, 9.6 ± 0.9 and 10.6 ± 3.2 μg/mL for β-elemene given by a continuous infusion pump over a period of 3 h in the dose groups of 10, 15, and 20 mg/kg [29]. The median exposure concentration of β-elemene measured in this study was lower than that measured by Chen et al. This may be due to differences in the mode and timing of administration. However, our study only collected the plasma sample at peak time, which could not calculate the pharmacokinetic parameters. Furthermore, as Figure 4 illustrates, there was no significant difference in β-elemene exposure level between the two groups (*p*=0.97, 95% CI: −3902.3–4232.0, student-*t* test), which may also be related to the short half-life of β-elemene, its rapid metabolism *in vivo*, and its use in combination with other antineoplastic agents. Further study with more strict inclusion and exclusion criteria and a larger sample size may benefit the assessment of exposure–effect of β-elemene.

## 4. Conclusion

The study successfully established and validated a GC–MS method for the quantitative analysis of β-elemene in human plasma, characterized by high sensitivity, a short total run time, and a simple and highly efficient sample pretreatment method. This method effectively quantified β-elemene concentrations in the plasma of 73 cancer patients. Notably, the *in vivo* exposure levels of β-elemene may not correlate with treatment efficacy. Additional studies with larger sample sizes and more stringent inclusion and exclusion criteria are necessary to further substantiate these findings. Besides, investigations of potential biomarkers based on, for example, metabolomics and epidemiological data of β-elemene may further promote its individualized application.

## Figures and Tables

**Table 1:** Linearity parameters of the β-elemene.

Analyte	Linear range (ng/mL)	Regression equation	*R* ^2^	Weighing factor
β-elemene	200.00–20,000.00	*y* = 2.7653*x* + 130.89	0.99	1/*χ*^2^

**Table 2:** Intra- and interday precision and accuracy of the analyte in human plasma (*n* = 3).

Analyte	Concentration (ng/mL)	Intraday			Interday		
		Measured concentration ± SD (ng/mL)	Precision (RSD%)	Accuracy (RE%)	Measured concentration ± SD (ng/mL)	Precision (RSD%)	Accuracy (RE%)
β-elemene	200.00	211.71 ± 3.98	1.90	5.86	213.20 ± 5.78	2.70	6.60
	400.00	358.48 ± 6.24	1.70	−10.38	374.85 ± 21.86	5.80	−6.29
	4000.00	3849.82 ± 22.98	0.60	−3.75	3865.80 ± 74.52	1.90	−3.35
	16000.00	14,583.92 ± 166.73	1.10	−8.85	14,683.75 ± 143.55	1.00	−8.23

**Table 3:** Matrix effects and extraction recoveries of the analyte in human plasma (*n* = 6).

Analyte	Concentration (ng/mL)	Recovery		Matrix Effect	
		Mean % ± SD	RSD%	Mean % ± SD	RSD%
β-elemene	400.00	94.89 ± 3.74	3.94	98.41 ± 0.18	0.19
	4000.00	96.25 ± 0.26	0.27	100.15 ± 1.88	1.88
	16000.00	87.95 ± 0.52	0.59	107.48 ± 0.77	0.72

**Table 4:** Stability of the analyte in human plasma (*n* = 3).

Analyte	Concentration (ng/mL)	Freeze–thaw stability		Ambient temperature 6 h		Long-term stability (−80°C, 1 months)		Autosampler at 4°C for 6 h	
		Precision (RSD%)	Accuracy (RE%)	Precision (RSD%)	Accuracy (RE%)	Precision (RSD%)	Accuracy (RE%)	Precision (RSD%)	Accuracy (RE%)
β-elemene	400.00	5.00	−5.40	0.90	−9.16	0.50	−7.50	1.70	−10.38
	4000.00	1.40	−2.82	0.90	−3.69	0.70	−2.29	0.60	−3.75
	16000.00	1.10	−8.28	0.20	2.56	2.20	−8.33	1.10	−8.85

**Table 5:** Clinical characteristics of enrolled cancer patients.

Categorization	*n* (%)
*Gender*	
Male	51 (70%)
Female	22 (30%)

*Age*	
≥ 60	36 (49%)
< 60	37 (51%)

*Cancer grading*	
I	2 (3%)
II	10 (14%)
III	16 (22%)
IV	45 (62%)

*Treatment outcome*	
PD	19 (26%)
SD	49 (67%)
Death	1 (1%)
PR	4 (5%)

*Therapeutic regimen*	
Radiotherapy	17 (23%)
Radiotherapy + immunotherapy	9 (12%)
Chemotherapy + targeted	30 (41%)
Chemotherapy + immunotherapy + targeted	9 (12%)
Target	2 (3%)
Targeted + immunity	6 (8%)

*Primary site*	
Malignant tumor of the colon	24 (33%)
Malignant tumor of the rectum	19 (26%)
Malignant tumor of the stomach	7 (10%)
Malignant tumors of the lungs	6 (8%)
Malignant tumors of the esophagus	4 (5%)
Malignant tumors of the breast	3 (4%)
Malignant tumor of the liver	1 (1%)
Malignant tumor of the ileocecal region	1 (1%)
Tumors of the urinary system	1 (1%)
Malignant tumor of the ovary	1 (1%)
Malignant tumor of the tongue	1 (1%)
Malignant tumor of the gallbladder	1 (1%)
Dedifferentiated liposarcoma	1 (1%)
Peritoneal mesothelioma	1 (1%)
Duodenal mesothelioma	1 (1%)
Neuroendocrine tumor	1 (1%)
Malignant tumors of the oral cavity	1 (1%)

*Pathological diagnosis*	
Squamous carcinoma	6 (8%)
Adenocarcinoma	60 (82%)
Ductal carcinoma	1 (1%)
Small cell carcinoma	6 (8%)

## Data Availability

The data that support the findings of this study are available from the corresponding author upon reasonable request.

## Supporting Information

Detailed methods for the LC-MS/MS method validation.
