# Supplementary material for: Quantification of β-Elemene by GC–MS and Preliminary Evaluation of Its Relationship With Antitumor Efficacy in Cancer Patients
Source: J Anal Methods Chem. 2025 Mar 30;2025:6694947. doi: 10.1155/jamc/6694947 (PMC11972858; doi:10.1155/jamc/6694947)
Supplement: Supporting Information — Additional supporting information can be found online in the Supporting Information section. [file 6694947.f1.docx]

**Supplementary materials:**

Detailed methods for the LC-MS/MS method validation:

1. Specificity and carryover

The evaluation of specificity was usually completed by comparing the responses of analytes in at least 6 different lots of spiked sample, blank samples and real samples, and it was considered reasonable that the responses of analytes in the blank samples not exceed 20% of the Lower Limit of Quantification (LLOQ) sample and 5% of the IS. Carryover in the GC-MS method is evaluated by injecting blank sample after the highest calibration standard for 3 cycles, and the responses of the analytes in the blank sample should be less than 20% of the LLOQ sample and 5% of the IS.

2. Linearity and LLOQ

Calibration curves were regressed based on the corresponding nominal concentrations of analytes versus IS-corrected peak areas in at least 6 calibration standards using a least squares model. At least 3 analytical batches on different days (at least 2 days) should be assessed and the deviations of back-calculated concentrations in their corresponding curves should be within ± 15%, and for the LLOQ, which was the lowest point in the calibration curve, the deviations should be within ± 20%.

3. Inter- and intra-day precision and accuracy

The inter- and intra-day accuracy and precision were evaluated at 4 concentration levels (high, medium, low, LLOQ) with 5 replicates in each concentration level along with the calibration curves evaluation. Relative standard errors (RSD%) of no more than 15% for intra-day and inter-day precision will be acceptable(no more than 20% for LLOQ), and the relative errors (RE%) of intra-day and inter-day accuracy should be within ±15%(within ±20% for LLOQ).

4. Extraction recovery and matrix effect

Extraction recoveries and matrix effects were evaluated with six replicates of QC samples at three concentration levels (high, medium and low). The extraction recovery was the ratio of the peak area of the spiked sample to that of the post-extracted spiked sample at the same concentrations, and the matrix effect was the ratio of the peak area of the post-extracted spiked sample to that of the solvent-substituted sample at the same concentration.

5. Stability

Stability was evaluated with three concentration levels (low, medium and high) using QC samples under different conditions. In this study, the room temperature stability (6 h at 25°C), short-term stability (24 h in an autosampler), long-term stability (1-3 months at -80 °C), and three freeze-thaw cycle stability were assessed. A calibration curve was constructed when measurements were performed on the long-term stability samples. The deviations of the QC samples from the nominal concentrations under different conditions should be within ±15%.
